# Oxygen Delivery and Oxygen Consumption in Pediatric Fluid Refractory Septic Shock During the First 42 h of Therapy and Their Relationship to 28-Day Outcome

**DOI:** 10.3389/fped.2018.00314

**Published:** 2018-10-23

**Authors:** Chulananda D. A. Goonasekera, Joseph A. Carcillo, Akash Deep

**Affiliations:** ^1^Department of Anesthetics, King's College Hospital, London, United Kingdom; ^2^Divison of Pediatric Critical Care Medicine, University of Pittsburgh School of Medicine, Pittsburgh, PA, United States; ^3^Paediatric Intensive Care Unit, King's College Hospital, London, United Kingdom

**Keywords:** septic shock, children, oxygen delivery, oxygen consumption, mortality

## Abstract

**Background:** In septic shock, both oxygen delivery (DO_2_) and oxygen consumption (VO_2_) are dysfunctional. The current therapeutic regimens are geared to normalize global oxygen delivery (DO_2_) to tissues via goal directed therapies but mortality remains high at 10–20%.

**Methods:** We studied cardiac index (CI), systemic vascular resistance index (SVRI), central venous oxygen saturation (ScvO2), central venous pressure (CVP), peripheral oxygen saturation (SpO2), mean blood pressure (MBP), body temperature, blood lactate, base excess and hemoglobin concentration (Hb) in a cohort of children admitted in “fluid-refractory” severe septic shock to pediatric intensive care, over 4.5-years. We calculated their 6 h global oxygen delivery (DO2) and global oxygen consumption (VO2) over the first 42 h and looked at factors associated with VO2/DO2 ratio (i.e., global oxygen extraction, gO2ER) and 28-day mortality.

**Results:** Sixty-two children mean age (SD) 7.19 (5.44) years were studied. Fifty-seven (93%) children were sedated and mechanically ventilated and all received adrenaline or noradrenaline or both and added milrinone in 6 (9.6%). At 28 days, 9 (14.5%) were dead. The global oxygen extraction ratio (gO2ER) was consistently lower amongst the survivors and independently predicted mortality (ROC AUC = 0.75). A lactate level of 4 mmol/l or above, when associated with a concurrent metabolic acidosis predicted mortality with a sensitivity of 100% (95% CI 90.5–100) and a specificity of 67.7% (95% CI 62.2–72.9). A gO2ER of 0.48 or above on admission to the PICU was associated with death with a 66.7% sensitivity (95%CI 29.9–92.5) and 90.5% specificity (95%CI 79.3–96.8). A global O2ER of >0.48 combined with a concurrent blood lactate >4.0 mmol/l at any time within the first 42 h of therapy predicted death with a sensitivity of 63.9% (95% CI, 46.2–79.1) and specificity of 97.8% (95% CI, 95.7–99.0). A radar plot identified MBP-CVP difference, and CI as additional goals of therapy that may offer a survival benefit.

**Conclusions:** Global O2ER of >0.48 with a concurrent blood lactate >4.0 mmol/l in children with metabolic acidosis was an independent factor associated with death in fluid resistant septic shock. Trends of gO2ER seem useful to recognize survivors and non-survivors early in the illness.

## Background

Childhood mortality in invasive infections, sepsis and septic shock remains high at 3.9, 5.6, and 17.0%, respectively (1, 2). Significant advances in the management of septic shock in children have been made and it hails from standardized, timely, goal directed therapies to normalize “macro-circulation” abnormalities (3). Clinicians target global oxygen delivery (DO_2_) as the therapeutic goal with the hope of matching the supply-demand gap of oxygen during the treatment of shock.

In septic shock, both oxygen delivery and oxygen consumption are dysfunctional. Some authors claim normalizing ScvO_2_ as an additional goal of therapy may be beneficial (4) whilst others argue that higher than normal ScvO_2_ may increase the mortality risk (5, 6). Higher ScvO_2_ can indicate improved oxygen delivery but it does not rule out continuing hypoxia (7). This is because high ScvO_2_ can also reflect poor tissue oxygen consumption resulting from sepsis induced dysfunctional capillary perfusion and altered mitochondrial function. However, quantitative estimations of these are scant.

Microcirculatory alterations in sepsis are associated with organ dysfunction and mortality (8, 9). Due to practical difficulties of measuring regional oxygen consumption and oxygen delivery, most clinical studies have focused on global oxygen delivery (DO_2_) and global oxygen consumption (VO_2_). Oxygen delivery is formally calculated using the degree of hemoglobin (Hb) oxygen saturation, and dissolved O_2_ content in arterial blood and cardiac output (CO). The oxygen consumption (VO_2_) is a composite estimate of global oxygen utilization. It is the calculated oxygen content difference between arterial and venous blood standardized to cardiac index.

To understand the evolution of DO_2_ and VO_2_ in pediatric “fluid resistant” septic shock during the resuscitation and stabilization phase and its associations, we estimated global oxygen delivery (DO_2_) and global oxygen consumption (VO_2_) and outcomes in a cohort of children in “fluid resistant” septic shock who were receiving standard goal directed therapy in the pediatric intensive care.

## Methods

We retrospectively studied hemodynamic variables during initial stabilization for 42 h and 28-day outcome in all children admitted from June 2009–Feb 2014 in fluid refractory septic shock to a regional 16-bed Pediatric Intensive Care Unit (PICU). Shock was classified as “fluid-refractory” when the total fluid requirement during resuscitation exceeded 60 ml/kg fulfilling international consensus conference criteria (10, 11) and required inotrope/vasopressor/vasodilator therapy.

All children had continuous hemodynamic monitoring including invasive arterial blood pressure and central venous pressure (via SVC catheter) as standard. Vasoactive agents (noradrenaline, adrenaline or milrinone) were used to optimize hemodynamics as per the ACCM-CPP protocol (12). In addition, their cardiac index (CI) and systemic vascular resistance index (SVRI) were measured approximately 6-h by an Ultrasonic Cardiac Output Monitor (USCOM model 1-A, USCOM Limited Australia http://www.uscom.com.au/) using a trans-cutaneous Doppler probe placed at the supra sternal notch (13, 14).

We calculated global oxygen delivery (DO_2_) using a standard formula (Equation 1) using pulse oximetry (SpO_2_) as an suitable surrogate for oxygen saturation of arterial blood (SaO_2_) (15). DO_2_ was standardized to body surface area (DO_2_I).

Equation 1 Oxygen delivery index (DO_2_I) calculation formula (normal range 520-720 ml O_2_/min/m^2^).

DO_2_I = O_2_ delivery index = DO_2_ mls/min/m^2^ = (10 × Hb/dl × 1.34 × SpO_2_) + (PaO_2_ in mmHg × 0.003 × 10) × Cardiac index l/min/m^2^.

We also obtained simultaneous measured central venous oxygen saturations (ScvO_2_) (16–18) of venous samples obtained via radiologically confirmed central venous catheters placed in superior vena cava (SVC) and estimated mixed venous oxygen content. Global oxygen consumption (VO_2_) was derived by subtracting estimated oxygen content in mixed venous blood from that of arterial blood (Equation 2). This too was standardized to body surface area (VO_2_I).

Equation 2 Oxygen consumption index (VO_2_I) calculation formula (normal range 110–160 ml O_2_/min/m^2^).

VO_2_I = Oxygen consumption mls/min/m^2^ = [(SpO_2_ – ScvO_2_) × 10 × 1.34 × Hb/dl] + [(PaO_2_ - PvO_2_ in mmHg) × 0.003 × 10] × Cardiac index l/min/m^2^.

We used the ratio of global oxygen consumption (VO_2_) to global oxygen delivery (DO_2_) standardized to body surface area i.e., VO_2_I/DO_2_I ratio (global oxygen extraction ratio - gO_2_ER, normal 0.28) as a crude clinical measure of global “efficacy” of oxygen consumption during resuscitation and stabilization phases of sepsis. We contemplated that global “efficacy” depended upon a combination of factors including the ability of tissues to extract oxygen and the reserve capacity of the cardiovascular system to increase oxygen delivery to match the peripheral oxygen demand. A higher gO_2_ER value was therefore suggestive of inadequate oxygen delivery (DO_2_) or increased oxygen consumption (VO_2_) or both. A lower gO_2_ER was reflective of increased oxygen delivery or decreased oxygen consumption (VO_2_) or both.

### Statistics

Absolute values were standardized to body surface area to adjust for age as appropriate. Continuous variables were summarized as mean (SD) and categorical data as count (percentage). Student *t*-test was used to test differences in continuous variables and the χ^2^ test to compare proportions. All analyses were performed with statistical software IBM-SPSS version 20 (IBM Corporation, New York, USA). All tests were 2-tailed, and *P* < 0.05 was considered statistically significant.

Using gO_2_ER values, a receiver operating characteristic curves (ROC) was developed against outcome. The area under the curve (AUC) of more than 0.75 was considered clinically useful (19).

To have an “aerial view,” we also created radar plots of the above parameters observed over the first 42 h for survivors and non-survivors separately. These plots represented mean ± SE of each variable to enable a visual comparison between surviving and non-surviving populations.

## Results

There were 62 children (M: F = 34:28) aged mean (SD) 7.19 (5.44) years admitted in fluid refractory septic shock. Nine required continuous veno-venous hemofiltration (CVVH) during ICU stay. Upon admission to the PICU, 93.5% children were mechanically ventilated and sedated using a combination of morphine and midazolam. At 42 h 85% of the surviving children were still on ventilators. A demographic difference between survivors and non-survivors was not observed but the PIM2 score was worse as expected in non-survivors (Table 1).

**Table 1:** Demographic data of survivors vs. non-survivors (*t-test, χ^2^ chi sq, ^U^Mann Whitney *U*-test).

	**Survivors**	**Non-survivors**	***P*-value**
*n*	53 (85.4%)	9 (14.6%)	
Age yrs. mean (*SD*)	6.87 (5.49)	9.0 (5.04)	0.27*
Gender M:F	31:22 (58% male)	3:6 (33% male)	0.1χ^2^
Hospital vs. community acquired infection	33:20 (62% hospital acquired)	5:4 (55% hospital acquired)	0.7χ^2^
Co-morbidities	None 29, liver transplant 6, cerebral palsy 4, other syndromic 4, liver disease 3, leukemia 3, bone marrow transplant 2, sickle cell disease 1, urea cycle disorder 1	None 3, liver transplant 1, leukemia 1, sickle cell disease 1, urea cycle disorder 1, cystic fibrosis 1, muscular dystrophy 1	
On Immunosuppression (steroids or tacrolimus)	17 (32%)	4 (44%)	0.47χ^2^
Ventilated on admission	51 (96%)	9 (100%)	0.5χ^2^
CVVH (n)	6 (11%)	3 (33%)	0.08χ^2^
PIM2 score mean(SD)	31.33 (29.08)	54.47 (32.11)	0.03*
PICU stay median (range) days	6 (2–122)	2 (0–20)	0.002^U^

Fifty-nine (95%) had a positive bacterial blood culture (Vancomycin Resistant E coli 2, Pneumococcal 3, Staph Aureus 12, MRSA 1, Pseudomonas 4, Coag Neg staph 7, E coli 10, Enterococcus 2, Streptococcus 7, Klebsiella 8, and Meningococcal 3). At 28 days, 9 (14.5%) children were dead. Of these, 6 (2/3rd) died within the first 42 h of admission to the PICU. Fourteen (26%) of the survivors and 4 (44%) of the non-survivors had a Hickman line *in situ*.

**Figure 1:**
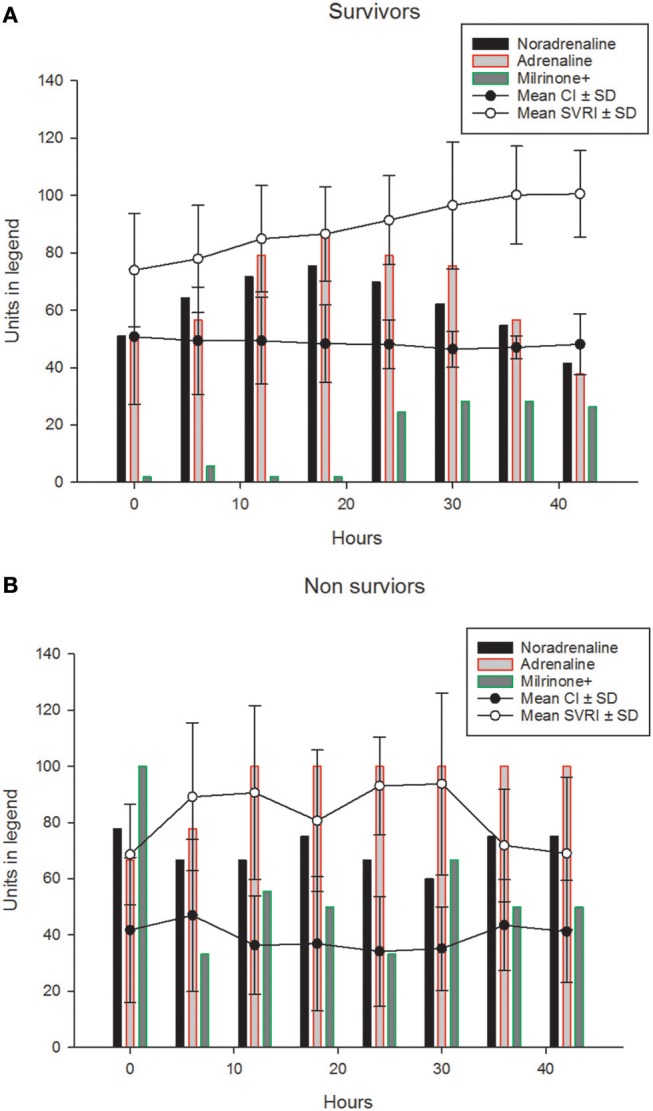
The (mean ±*SD*) progression of CIx10 l/ min/m^2^ (line graph) and SVRI÷10dyne s/cm^5^/m^2^ (line graph) and the percentages of children (bar graphs) receiving, adrenaline, noradrenaline and milrinone (±other vasopressors such as Terlipressin) during the first 42 h in **(A)** survivors. The (mean ±*SD*) progression of CIx10 l/min/m^2^ (line graph) and SVRI÷10dyne s/cm^5^/m^2^ (line graph) and the percentages of children (bar graphs) receiving, adrenaline, noradrenaline and milrinone (±other vasopressors such as Terlipressin) during the first 42 h are shown in **(B)** non-survivors.

**Figure 2:**
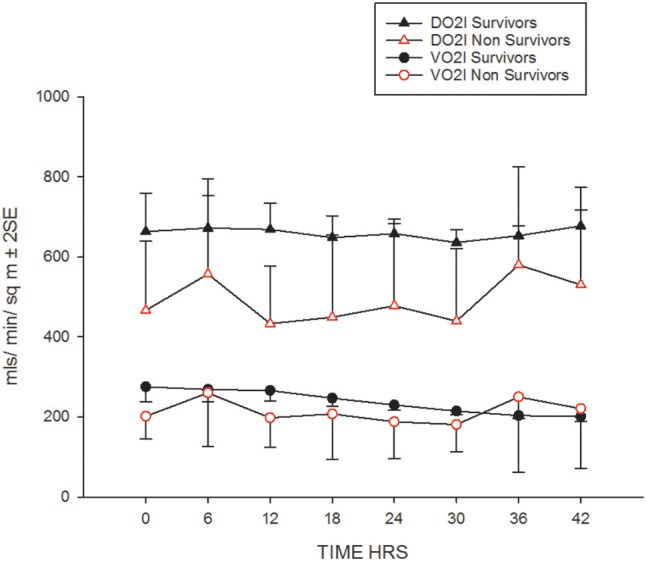
The evolution of DO_2_I and VO_2_I in the first 42 h.

**Figure 3:**
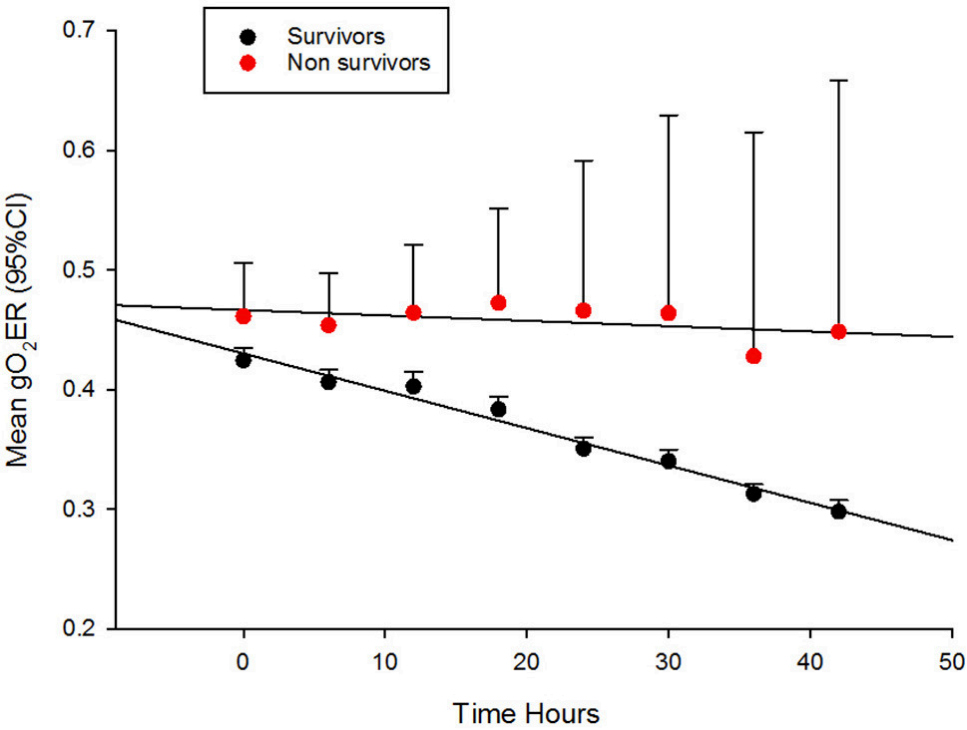
The evolution of global oxygen extraction ratio (gO_2_ER) in fluid refractory septic shock in children during the first 42 h of therapy in PICU.

Their progression of CI, SVRI and the use of inotropes and vasopressors are shown in Figures 1A,B and Table 2. The CI values during the 42-h study period on average were significantly higher amongst survivors compared to non-survivors [mean (SD), 4.8 (1.4) vs. 3.9 (2.1) l/min/m^2^, ANOVA F = 16.8, *p* < 0.001]. The SVRI values recorded during the 42 h study period were also significantly higher amongst survivors compared to non-survivors [mean (SD), 889.9 (201.6) vs. 827.8 (254.9) dyne s/cm^5^/m^2^, ANOVA F = 4.2, *p* < 0.03]. The evolution trends of DO_2_I and VO_2_I are shown in Figure 2. The mean DO_2_I was consistently above 600 ml/min/m^2^ in survivors [mean (SD), 659.5 (210.9) vs. that of non-survivors 485.3 (255.5) mls/min/m^2^, ANOVA F = 30.5, *p* < 0.001]. The concurrent VO_2_I reduced with time in survivors (Figure 2). The initial gO_2_ER was elevated in all (expected normal 0.25 – 0.3) but gradually reduced with minimal variability in survivors. In contrast, the gO_2_ER remained high amongst non-survivors (Figure 3). The widening variability was due to loss of numbers with time. The gO_2_ER correlated with SpO_2_-ScvO_2_ gap; *r* = 0.98 (*P* < 0.0001) and *r* = 0.93 (*p* < 0.0001) amongst survivors and non-survivors respectively.

**Table 2:** Six hourly cardiovascular parameters of survivors (s) and non-survivors(n-s) described in median (range) (1 cmH_2_O = 0.735 mmHg).

**CVS variable**	**0 h**		**6 h**		**12 h**		**18 h**		**24 h**		**30 h**		**36 h**		**42 h**	
	**(s)**	**(n-s)**	**(s)**	**(n-s)**	**(s)**	**(n-s)**	**(s)**	**(n-s)**	**(s)**	**(n-s)**	**(s)**	**(n-s)**	**(s)**	**(n-s)**	**(s)**	**(n-s)**
CI l/min/m^2^	3.2 (2.5–8.9)	2.9 (2.0–8.3)	4.5 (2.6–8.4)	3.4 (2.0–9.3)	5.0 (2.7–8.7)	3.0 (1.6–6.2)	4.8 (2.7–8.8)	2.7 (1.3–8.3)	4.8 (3.0–7.0)	3.1 (1.0–6.0)	4.7 (2.0–6.0)	4.2 (2.0–5.0)	4.8 (3.0–6.0)	4.9 (2.0–6.0)	4.7 (4.0–12.0)	4.7 (2.0–6.0)
SVRI dyne s/cm^5^/m^2^	732 (423–1433)	656 (432–900)	782 (408–1150)	912 (341–1207)	812 (450–1500)	821 (456–1487)	887 (351–1286)	787 (456–1154)	899 (427–1346)	958 (712–1198)	912 (678–2311)	888 (555–1453)	987 (776–1770)	766 (454–889)	987 (874–1827)	765 (321–910)
MBP–SVP (mmHg)	39.6 (24.1–57.9)	33.3 (28.6–50.3)	42.9 (28.1–58.9)	40.9 (34.6–52.3)	44.9 (11.5–58.9)	43.1 (29.6–57.6)	48.9 (33.3–69.3)	41.3 (26.9–51.6)	51.6 (38.9–66.9)	43.3 (31.9–47.9)	54.2 (43.1–71.0)	44.3 (23.8–50.7)	59.1 (38.6–72.9)	43.9 (33.2–53.7)	59.9 (41.4–73.6)	42.1 (37.1–50.2)
DO_2_I (ml/min/m^2^)	497.1 (257.8–1557.2)	364.6 (191.7–889.3)	582.0 (263.6–1282.0)	396.4 (231.5–1276.2)	580.5 (245.0–1202.2)	382.6 (177.6–802.4)	638.4 (343.3–1124.5)	335.5 (126.1–953.6)	621.7 (451.4–929.9)	463.3 (198.9–716.7)	629.4 (314.6–875.1)	543.3 (165.7–643.6)	663.9 (399.8–846.7)	636.0 (235.5–812.3)	655.3 (514.5–1505.6)	640.2 (168.3–672.6)
VO_2_I (ml/min/m^2^)	237.7 (110.1–672.1)	184.7 (102.4–359.1)	249.6 (123.0–517.1)	191.9 (102.4–727.0)	240.8 (135.2–546.5)	147.6 (102.4–429.1)	226.0 (130.1–436.8)	163.5 (077.2–577.6)	221.3 (147.3–360.3)	153.4 (074.5–397.1)	213.8 (131.2–286.9)	162.2 (118.1–309.0)	208.9 (128.8–261.7)	173.9 (120.6–530.1)	193.2 (154.2–435.2)	171.2 (101.6–438.7)
gO_2_ER	0.42 (0.35–0.51)	0.48 (0.35–0.53)	0.40 (0.32–0.48)	0.43 (0.38–0.57)	0.40 (0.25–0.55)	0.47 (0.35–0.59)	0.38 (0.31–0.58)	0.46 (0.31–0.61)	0.34 (0.27–0.45)	0.47 (0.31–0.65)	0.33 (0.29–0.46)	0.47 (0.28–0.71)	0.31 (0.25–0.40)	0.39 (0.27–0.65)	0.30 (0.25–0.39)	0.45 (0.24–0.65)

**Figure 4:**
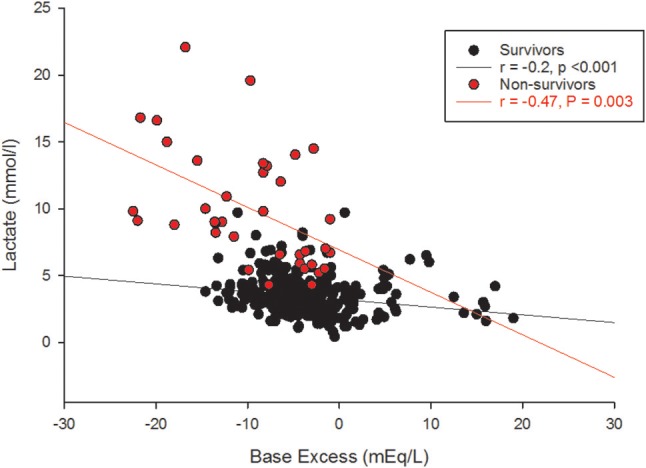
Lactate and base excess amongst survivors and non-survivors.

The receiver operating characteristic (ROC) curve of the 6 h gO_2_ER values of all children observed during the first 42 h showed an area under the curve (AUC) of 0.76 against death. A gO_2_ER of 0.48 or above on admission to the PICU predicted death with a 66.7% sensitivity (95%CI 29.9–92.5) and 90.5% specificity (95%CI 79.3–96.8). The ROC curve of blood lactate levels of all children observed during the first 42 h at 6 hourly intervals against 28-day mortality also showed an AUC of 0.97. A lactate level of 4mmol/l or above, associated with a concurrent metabolic acidosis within the first 42 h of therapy linked to mortality with a sensitivity of 100% (95% CI 90.5–100) and a specificity of 67.7% (95% CI 62.2–72.9). However, the base excess alone was nonpredictive of the mortality risk (ROC AUC was 0.25) (see Figure 4).

A global O_2_ER of >0.48 combined with a concurrent blood lactate >4.0 mmol/l at any time within the first 42 h of therapy predicted death with a sensitivity of 63.9% (95% CI, 46.2–79.1) but a specificity of 97.8% (95% CI, 95.7–99.0). The gO_2_ER significantly positively correlated with blood lactate (survivors *r* = +0.68, *p* < 0.001, non-survivors *r* = +0.74, *p* = 0.003) and negatively with base excess in survivors (*r* = −0.2, *p* < 0.001) but not in non-survivors (*r* = −0.15, *p* = 0.3).

**Figure 5:**
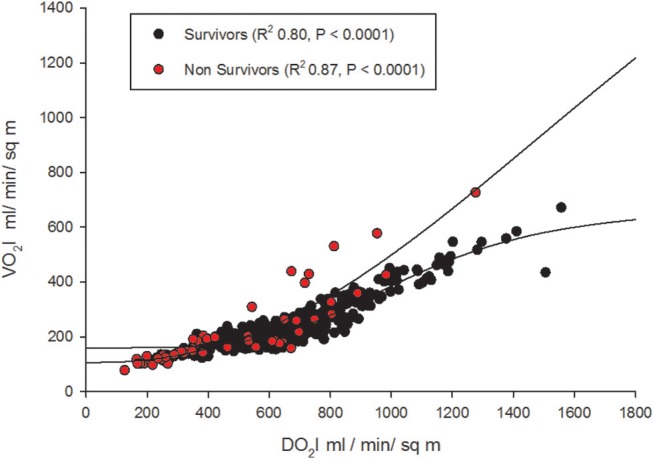
Oxygen supply-dependence graphs (with non-linear logistic regression curves) amongst survivors and non-survivors.

Oxygen supply dependence graphs were generated for survivors and non-survivors (Figure 5). A critical DO_2_ was not demonstrable in these curves. The SpO_2_-ScvO_2_ gap had a significant negative correlation with CI (*r* = −0.16, *p* = 0.001) and SVRI (*r* = −0.28, *p* < 0.001) in survivors. However, SpO_2_-ScvO_2_ gap neither correlated with CI (*r* = −0.07, *p* = 0.5) nor with SVRI (*r* = −0.17, *p* = 0.2) in non-survivors.

**Figure 6:**
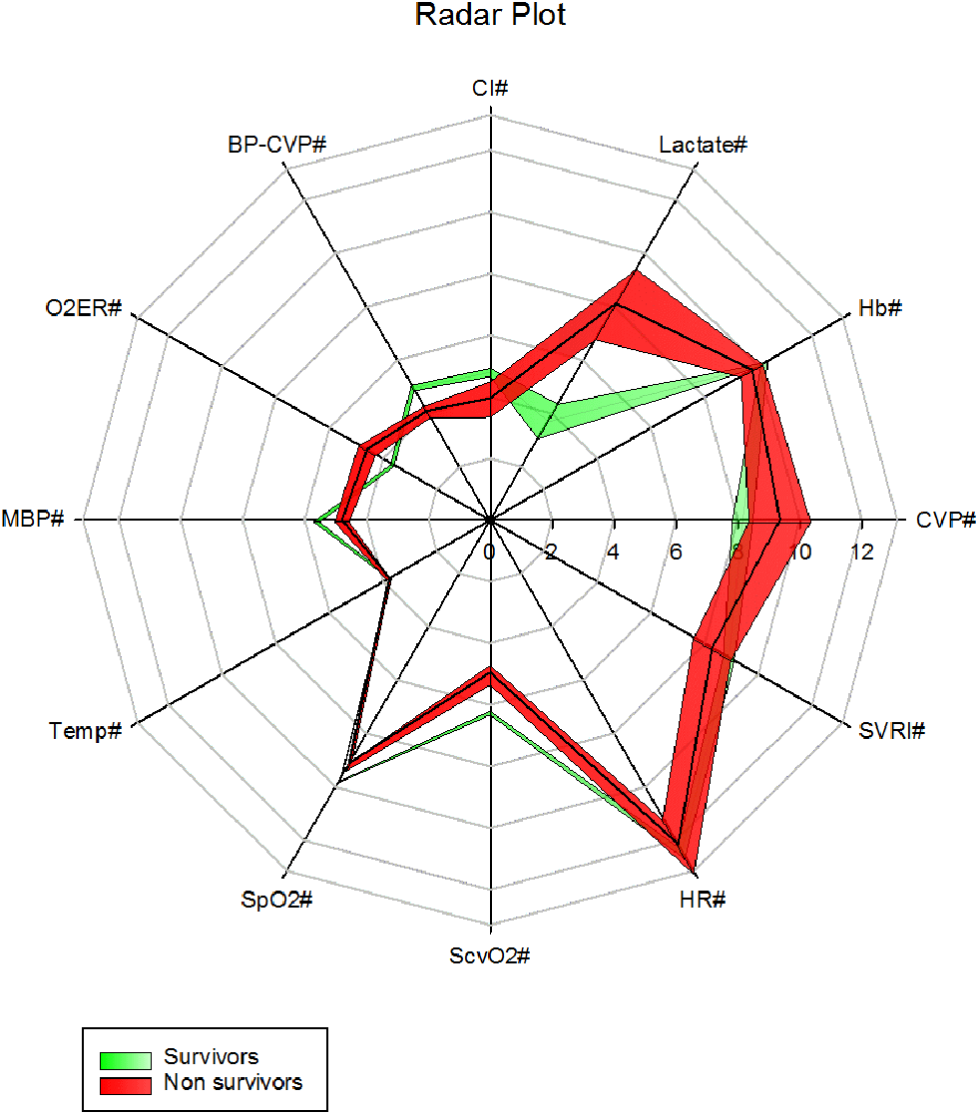
A radar plot showing mean ± 2SE area fills of 6 h first 42 h clinical parameters between survivors and non survivors (CVP# = CVP cmH_2_O, Hb# = Hb g/dl, Lactate# = Lactate mmol/l, CI# = CI l/min/m^2^, MBP-CVP# = [Mean BP- CVP/10]mmHg, gO_2_ER# = gO_2_ER x 10, MBP# = [mean BP/10] mmHg, Temp# = [Temperature /10]°C, SpO_2_# = [SpO_2_/10]%, ScvO2# = [ScvO2 x 10]%, HR# = [Heart rate/10] /min, SVRI# = [SVRI/100]dyne s/cm^5^/m^2^).

VO_2_I significantly positively correlated (2 tailed, Pearson) with CI (*r* = +0.817, *p* < 0.001) and negatively with SVRI (*r* = −0.542, *P* < 0.001). The overview radar plots of above clinical parameters drawn separately for survivors and non-survivors with the respective ± 2SE fills enabled visual recognition of the areas of disparity that needs attention to bring the non-survivor plot closer to that of survivors (Figure 6).

**Figure 7:**
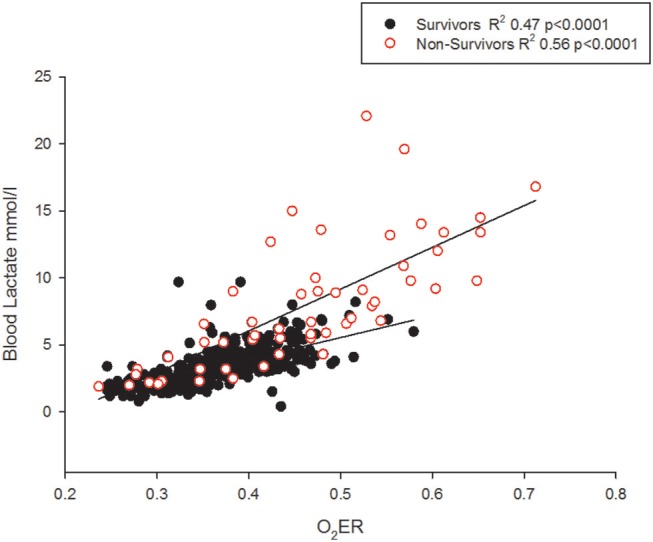
The O_2_ER and blood lactate correlate but non-survivors may demonstrate lower lactate levels.

Neither the Hb on admission nor the body temperature was associated with outcome. The O_2_ER correlated with blood lactate levels (Figure 7).

## Discussion

We have presented a comprehensive account of the cardiovascular parameters in a cohort of children in “fluid resistant” septic shock during the first 42 h of bundle therapy in intensive care. Their severity of illness is indicated by the PIM2 scores (20). The titration of inotropes and vasopressors during treatment was assisted by non-invasive 6 hourly cardiac output monitoring. The limitation of our study to 42 h was based on lack of data resulting from steadily receding need for inotropes (21) and clinicians deciding to taper off invasive monitoring and regular cardiac output measurements in children showing improvement. We also looked at the physiological variables that accounts for DO_2_ and its influence upon mortality and the reliability of ScvO_2_ as a therapeutic efficacy evaluation tool.

### Critical DO_2_

The expected normal values for global DO_2_I and global VO_2_I are 500 ml /min/m^2^, and 150 ml/min/m^2^ respectively in adults (22). In the new born DO_2_I is high at birth reducing to a mean (SD) of 609 (141) ml/min/m^2^ at 3 days of age (23). We noted that global DO_2_I above 600 ml/min/m^2^ during the first 42 h of therapy in PICU was more likely to be associated with survival (Figure 2) in our cohort. A trial involving adult high-risk surgical postoperative patients too has identified a global DO_2_I above 600 ml/ min/m^2^ as critical for survival (24).

We could not demonstrate a critical DO_2_ in our cohort, especially non-survivors (Figure 5). This refers to the point at which the compensatory increase in O_2_ extraction is exhausted with the lowering DO_2_, and the VO_2_ becomes dependent on DO_2_ (25). This pathologic O_2_ supply dependency possibly results from loss of efficient tissue distribution of DO_2_ (26). In fact, this abnormal dependence of VO_2_ on DO_2_ and the absence of a critical DO_2_, may be the “hallmark” of fluid refractory septic shock state. In other words, tissue oxygen consumption is not met by the delivered oxygen despite maximum extraction leading to a state of chronic tissue oxygen debt. The longer this persists; more likely the shock state becomes irreversible as we have noted in our *gO*_2_*ER* plot of non-survivors (Figure 3). In adults too, VO_2_ dependency on DO_2_ is considered a marker of septic shock (27). It seems that clinically deliverable ranges of global DO2 achieved via current bundle therapies were insufficient to rectify this state of VO_2_ dependence on DO_2_ in our non-survivors. This observation is in agreement with that of recent epidemiological data showing children who die early from refractory septic shock, usually die of cardiovascular demise (28).

### gO_2_ER

Upon admission to PICU, gO_2_ER was approximately 0.5 in most children (Figure 3). Thus, gO_2_ER was less sensitive in predicting mortality on admission. However, we noted a steady improvement in gO_2_ER toward normal levels (0.25–0.3) with a narrow 95% CI during the first 42 h of therapy along with a progressive reduction in global VO_2_I in survivors (Figure 2). In contrast, a persistently high mean gO_2_ER was observed in non-survivors (Figure 3) with a progressively widening 95% CI due to reduced number of data following the demise of some children. A gO_2_ER greater than 0.5 at 6 h was a recognized risk factor of death after cardiac operations in infants (29). We also observed that the first 42-h gO_2_ER value was independently associated with 28-day mortality (Figure 3).

In our cohort, a high gO_2_ER resulted from a disproportionately lower DO_2_ due to a lower CI especially amongst non-survivors (Figure 2). This might reflect an inability of the myocardium to respond to the demand i.e., a state of myocardial “stunning” alluded to mitochondrial dysfunction (30–32). Unmasking of myocardial weakness during treatment of septic shock is known (21). We observed that persistently high gO_2_ER and lack of attainment of critical DO_2_ in the initial phases of therapy may identify children in septic shock with chronic tissue oxygen debt progressing to an irreversible shock state and death. This suggests that early options of treatment above the standard is needed in the above sub-set of children, for example, inodilators such as milrinone or levosimendan, or even extracorporeal membrane oxygenation (ECMO) to attain critical DO_2_ and save life (33) (34). This is because our data suggest that the DO_2_ was far short of matching the oxygen need or consumption especially amongst non-survivors. In this context, gO_2_ER seems a valid and independent global estimate of the real time “efficacy” of O_2_ consumption in this clinical scenario and a tool to identify children at high risk of death although cellular oxygen uptake and utilization are not verifiable from global measurements (35).

### ScvO_2_

When DO_2_ is insufficient to meet metabolic requirements, increased tissue oxygen extraction reduces oxygen content in effluent venous blood (16). Thus, a high SpO_2_-ScvO_2_ gap is a crude estimate of high oxygen consumption at tissue level. This explains its crude use as a marker of inadequate tissue perfusion and a therapeutic goal in critical care practice. We observed a strong correlation between SpO_2_-ScvO_2_ gap and gO_2_ER as expected.

It is also contemplated that ScvO_2_ may read higher when O_2_ consumption is reduced at capillary level (16–18). In other words, SpO_2_-ScvO_2_ gap should be lower in the presence of arterio-venous (A-V) shunting i.e., in severe sepsis. On the contrary, we observed that SpO_2_-ScvO_2_ gap significantly correlated negatively with CI and SVRI in survivors, i.e., with clinical improvement. But, it neither correlated with CI nor with SVRI in non-survivors. This suggested that ScvO_2_ had little monitoring value in children in whom it matters most i.e., in non-survivors. Although ScvO_2_ has been shown to be useful in guiding resuscitation (36), its validity in the resuscitated subjects remains a matter of controversy (37). Further, a reduction in ScVO_2_ when CI was < 3.3 as well as > 6.0 (l/m^2^/min) has been observed in pediatric septic shock (21). This may also explain why we cannot overcome flow dependence as CI increases in our cohort (Figure 5).

### Lactate

Although lactate and base excess correlate, it's different between survivors and non-survivors (Figure 4). High lactate on hospital admission, and at 12 and 24 h in pediatric septic shock is reported to predict death (38). It appears that the risk of death was highest only when a metabolic acidosis (as detected by base excess) was present concurrently with a blood lactate level > 4 mmol (39). This is possibly because there were many other factors in addition to poor tissue perfusion contributing to the rise in blood lactate; for example, the use of adrenaline, renal impairment and liver dysfunction (40). This affirms that clinical and prognostic importance of an elevated lactate differs widely by disease state (39). In our study, gO_2_ER correlated positively with blood lactate (Figure 7), but lower levels of lactate amongst some non-survivors reduced its sensitivity in recognizing high mortality risk (Figure 7). This explains why a global O_2_ER of >0.48 combined with a concurrent blood lactate >4.0 mmol/l and a metabolic acidosis enhanced its sensitivity of assessing risk of death. The high O_2_ER together with metabolic acidosis makes the origin of lactate more likely from tissue anaerobic metabolism. Therefore, despite the confounding factors, we infer that gO_2_ER in combination with lactate can predict mortality with a significant sensitivity and specificity. However, negative base excess alone had no value in mortality prediction. Data to support or refute treatment of lactate acidosis with a buffer (sodium bicarbonate) or its clearance through dialysis in this scenario are scant (41).

### Radar plots

Our overview radar plot of cardiovascular variables was an attempt to recognize all vital parameters and therapeutic monitoring targets between non-survivors and survivors in a single figure (Figure 6). The use of mean ± 2 SE fills was an attempt to recognize what parameters may be significantly apart between the two populations i.e., surviving and non-surviving children presenting in fluid refractory septic shock. We presume that closing the gap in these parameters toward that of the survivors may improve chances of survival. The radar plot suggested areas of therapy that may be optimized. For example, increasing mean MBP-CVP gap by reducing the upper limit of CVP to 8 cm H_2_O, enhancing the lower limit of SVRI and increasing the cardiac index (by mechanical means) could be lifesaving if achieved concurrently. We have learned that fluids in excess of 40 mls/kg in resuscitation may improve survival in septic shock in children (42) but, overzealous use may be detrimental (43). A CVP of 8 cm H_2_O or below has been linked to lowest mortality in septic shock (43).

### Limitations of this study

The retrospective nature of our study limits its value, but all data used in this study has been prospectively collected in real-time and validated (i.e., authenticated and saved by the supervising nurse daily) and archived electronically in the “MetaVision” (Clinical Information System) database of the PICU. Thus, inherent errors of retrospective clinical studies based on paper records were minimized.

Another limitation of our study was ScvO_2_. Historically, site dependent measurement errors have rendered ScvO_2_ less valuable. Since ScvO_2_ can vary due to preferential blood flow patterns of superior vena cava (SVC) and inferior vena cava (IVC) (17, 44) and admixture of highly desaturated coronary blood flow, the ScvO_2_ may not reflect true oxygen consumption. In children with septic shock, low CI is generally associated with lower ScvO_2_; however, ScvO_2_ may also remain less than 70% in normal and in high cardiac output states (e.g., in warm shock) (21).

Similarly, the use of peripheral perfusion dependent SpO_2_ as a surrogate marker of SaO_2_ could have underestimated the DO_2_I especially in cold shock states. However, the margin of error ensued is likely to be very small as our mean (SD) SpO_2_ was 96.1 (3.6). On the other hand, the use of ScvO_2_ in the calculation of VO_2_I may also have added a margin of error. ScvO_2_ is more variable than SvO_2_ and tends to be an over-estimate in critically ill patients (45). It is also observed to remain less than 70% in some children with normal and high CI in septic shock (21). Although ScvO_2_ is useful in guiding resuscitation (36), its validity in the resuscitated is controversial (37).

CI measurement by USCOM is also subject to wide variation. In order to minimize inter-observer variability, USCOM operators were certified competent by the author (AD) as advocated by previous researchers (46).

In severe sepsis states, changes in ScvO_2_ due to preferential blood flow pattern and microcirculatory shunting at tissue level are both contributors to gO_2_ER. Therefore, the persistently high gO_2_ER that we observe in this study as a predictor of death is a global crude reflector of not just oxygen consumption, but also inherent circulation abnormalities associated with severe sepsis including microcirculatory shunting.

A drawback is that gO2ER cannot be used worldwide as CI is not measured at all PICUs and CI measurements have its own limitations (47, 48). However, our findings support its use in very specific conditions such as septic shock. This is in line with surviving sepsis guidelines emphasizing the early use of age-specific therapies to attain time-sensitive goals, specifically in the first hour of fluid resuscitation and inotrope therapy, and subsequent intensive care unit hemodynamic support (12).

## Conclusion

We observe that absolute ScvO_2_, and base excess were less robust indicators of outcome during initial 42 h of therapy of “fluid resistant” pediatric septic shock. In contrast, global O_2_ER of >0.48 found concurrently with a blood lactate >4.0 mmol/l and metabolic acidosis seems a better independent predictor of death.

We conclude that trends of gO_2_ER may recognize survivors and non-survivors early in the illness. Although, further studies are required to assess the validity of this bedside measure, a non-improving gO_2_ER may justify implementation of extraordinary measures of therapy such as mechanical cardiac support and novel pharmacologic strategies early in pediatric septic shock.

## Ethics statement

This was an observational study of routinely monitored clinical parameters in septic shock in the PICU. No patient identifiable data was stored. Therefore, according to the local guidance the study was registered as a service evaluation project at King's College Hospital, London (Clinical Audit Support System (CASS) project no. 2902).

## Author contributions

All authors contributed to manuscript revision, read and approved the submitted version. AD led collection of data; CG performed the statistical analysis, prepared figures and tables and wrote the first draft of the manuscript and managed revisions. JC assisted interpretation of data in the clinical context and previous research findings.

### Conflict of interest statement

The authors declare that the research was conducted in the absence of any commercial or financial relationships that could be construed as a potential conflict of interest.

## Abbreviations

95% CI: ninety five percent confidence interval
ANOVA: analysis of variance
AUC: area under the curve
A-V: arterio-venous
CI: cardiac index
CO: cardiac output
CVP: central venous pressure
CVVH: continuous veno-venous hemofiltration
DO_2_: global oxygen delivery
DO_2_I: global oxygen delivery index
ECMO: extracorporeal membrane oxygenation
gO_2_ER: global oxygen extraction ratio
Hb: hemoglobin
IVC: inferior vena cava
MAP: mean arterial pressure
MBP: mean blood pressure
PICU: Pediatric Intensive Care Unit
PIM2: Pediatric index of mortality 2
RBC: Red blood cell
ROC: receiver operating characteristic curve
SaO_2_: oxygen saturation arterial blood
ScvO_2_: central venous oxygen saturation
SD: standard deviation
SE: standard error
SpO_2_: peripheral oxygen saturation
SVC: superior vena cava
SVRI: systemic vascular resistance index
USCOM: Ultrasonic Cardiac Output Monitor
VO_2_: global oxygen consumption
VO_2_I: global oxygen consumption index
χ2: Chi square test.
